# Comparative Metabolomics Study of the Impact of Articaine and Lidocaine on the Metabolism of SH-SY5Y Neuronal Cells

**DOI:** 10.3390/metabo12070581

**Published:** 2022-06-23

**Authors:** Gustavo H. Rodrigues da Silva, Luís F. Mendes, Fabíola V. de Carvalho, Eneida de Paula, Iola F. Duarte

**Affiliations:** 1 CICECO—Aveiro Institute of Materials, Department of Chemistry, University of Aveiro, 3810-193 Aveiro, Portugal; gustavohrs@gmail.com (G.H.R.d.S.); luisfilipemendes@ua.pt (L.F.M.); 2 Department of Structural and Functional Biology, Institute of Biology, University of Campinas (UNICAMP), Campinas 13083-862, SP, Brazil; fabiolavieiracarvalho@hotmail.com (F.V.d.C.); depaula@unicamp.br (E.d.P.)

**Keywords:** local anesthetics, articaine and lidocaine, toxicometabolomics, neuronal cells, neurotoxicity

## Abstract

Articaine (ATC) and lidocaine (LDC) are the local anesthetics (LAs) currently most employed in dentistry. Cases of paresthesia, reported more frequently for ATC, have raised concerns about their potential neurotoxicity, calling for further investigation of their biological effects in neuronal cells. In this work, the impact of ATC and LDC on the metabolism of SH-SY5Y cells was investigated through ^1^H NMR metabolomics. For each LA, in vitro cultured cells were exposed to concentrations causing 10 and 50% reductions in cell viability, and their metabolic intracellular and extracellular profiles were characterized. Most effects were common to ATC and LDC, although with varying magnitudes. The metabolic variations elicited by the two LAs suggested (i) downregulation of glycolysis and of glucose-dependent pathways (e.g., one-carbon metabolism and hexosamine biosynthetic pathway), (ii) disturbance of branched chain amino acids (BCAA) catabolism, (iii) downregulation of TCA cycle anaplerotic fueling and activation of alternative energy producing pathways, (iv) interference with choline metabolism and (v) lipid droplet build-up. Interestingly, LDC had a greater impact on membrane phospholipid turnover, as suggested by higher phosphatidylcholine to phosphocholine conversion. Moreover, LDC elicited an increase in triglycerides, whereas cholesteryl esters accumulated in ATC-exposed cells, suggesting a different composition and handling of lipid droplets.

## 1. Introduction

**Figure 1:**
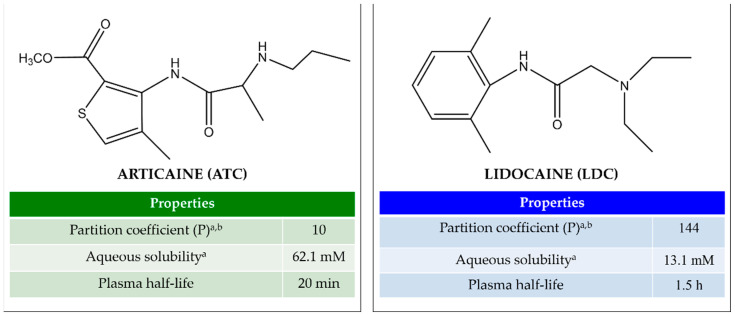
Molecular structures and main properties of articaine (ATC) and lidocaine (LDC) (ChemDraw 21.0 software, Perkin Elmer, MA, USA). ^a^ Determined for the neutral species; ^b^ determined between egg phosphatidylcholine/aqueous buffer [7].

Local anesthetics (LAs) are widely used in medical procedures for pain control, as they can interrupt the neuronal action potential, thus preventing the transmission of nociceptive information to the central nervous system [1]. In dental practice, the most employed LAs are amino-amide molecules, such as lidocaine (LDC, Figure 1A), considered the “gold standard” of local anesthesia [2,3], and articaine (ATC, Figure 1B), which has become increasingly popular in the last twenty years [4,5]. ATC has a unique structural feature consisting of a thiophene ring instead of a benzene ring. While this has been initially postulated to confer ATC increased lipophilicity [6], recent studies have shown that ATC has a 10× lower egg phosphatidylcholine/aqueous buffer partition coefficient (P) than LDC [7], and preferentially inserts in the polar head group of liposomes [8], a feature of more hydrophilic LAs [9]. Moreover, ATC has an ester group linked to the thiophene ring, which is quickly hydrolyzed by plasma esterases, making its plasma half-life very short (20 min) and diminishing its systemic toxicity (cardiac and neuronal) [10]. Due to this fact, ATC is formulated for infiltrative administration at higher doses (4%) than any other LA, either of low (e.g., LDC, mepivacaine and prilocaine: 2–3%) or high P (bupivacaine, ropivacaine, etidocaine: 0.5–0.75%) [7].

Adverse effects of dental LAs that are correctly administered have seldom been reported; hence, they are generally regarded as safe. However, there has been some concern about their potential neurotoxicity, raised mostly by cases of paresthesia, i.e., persistent nerve numbness or altered sensibility, which may be temporary or permanent [11]. Reports of paresthesia are more frequent for ATC than LDC or other LAs [12,13,14,15], raising up concerns about ATC local toxicity. Nevertheless, no evidence of a greater neurotoxicity of ATC in respect to other LAs was reported in the literature, either in vitro or in vivo [16,17,18], which shows the need of further research to elucidate this aspect.

Depending on the dose and exposure duration, all LAs elicit neurotoxic effects, which may contribute to perioperative neurological complications [19]. The cellular and molecular mechanisms of LAs’ neurotoxicity remain to be fully elucidated and may vary between LAs and cell types [19,20]. LDC has been reported to trigger mitochondrial injury and cell death in neuronal cells in a concentration-dependent manner [21,22,23]. For instance, LDC suppressed the electron transport chain (ETC) in SH-SY5Y neuroblastoma cells, causing attenuation of mitochondrial membrane potential and heightened production of reactive oxygen species (ROS), leading to apoptosis and necrosis [22]. The same authors further demonstrated that activation of the hypoxia-inducible factor 1 (HIF-1), involved in oxygen metabolism and mitochondria function, conferred resistance to LDC-induced apoptosis [24]. Furthermore, in primary sensory neuron cultures, LDC neurotoxicity was found to be mediated by lipoxygenase pathways downstream of specific activation of the p38 mitogen-activated protein kinase (MAPK), a serine–threonine kinase central to stress-induced apoptosis [25]. As for ATC, while its cytotoxicity towards neuronal cells has been assessed in a few studies [23,26,27], the subcellular mechanisms of ATC neurotoxic effects are still poorly defined and call for further research [16].

In this work, we have investigated the cytotoxicity and metabolic effects of both LDC and ATC in the SH-SY5Y cell line, derived from a human neuroblastoma. These cells express numerous dopaminergic neuronal markers, making its cytotoxic response similar to that of human primary neuronal cultures [28]. Moreover, SH-SY5Y cells express voltage-gated sodium channels (Na(v)1.2 and Na(v)1.7), which are targets for LA action [26], further supporting their relevance as a model in LAs neurotoxicity studies. To assess drug impact on cell metabolism, changes in the composition of cells and the extracellular medium were assessed through ^1^H NMR metabolomics, at concentrations inducing 10 and 50% decreases in cell viability. Metabolomics has been widely explored in the field of toxicology, including in studies aimed at assessing the neurotoxic effects of different drugs [29]. However, its application to the study of LAs has been limited to characterizing the effects of LDC in yeast [30] and in breast cancer cells [31]. To our knowledge, the work hereby presented is the first metabolomics report on the neurotoxicity of LAs. Our main objectives are (i) to assess the ability of LAs to modulate the central metabolism of neuronal cells, (ii) to determine the main metabolites and metabolic pathways affected by each studied anesthetic (LDC and ATC) and (iii) to formulate new hypotheses about LDC and ATC modes of action that might help to explain their cytotoxic profiles.

## 2. Results

### 2.1. Cell Viability

**Figure 2:**
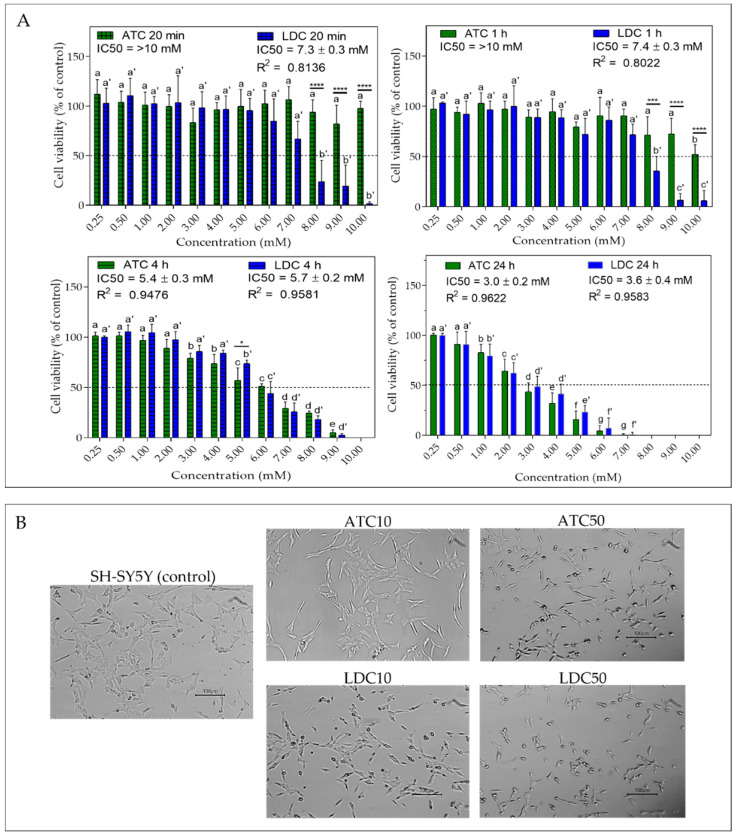
(**A**) Cell viability of SH-SY5Y neuroblastoma cells exposed to 0.25–10 mM of articaine (ATC) or lidocaine (LDC) after 20 min, 1 h, 4 h and 24 h of treatment. The IC50 values and the R^2^ values (reflecting the goodness of fit) are indicated. Statistical significance was assessed via one-way ANOVA; for each LA, significant differences between cell viabilities at different concentrations are indicated by different letters (without/with prime for ATC/LDC, respectively); significant differences between ATC and LDC, at each exposure concentration, are indicated by asterisks (* *p* < 0.05; *** *p* < 0.005; **** *p* < 0.001); (**B**) Micrographs of SH-SY5Y cells, without treatment (control) and after 24 h exposure to the local anesthetics at concentrations of IC10 (ATC10 and LDC10) and IC50 (ATC50 and LDC50). Scale Bar = 100 µm.

To assess the impact of ATC and LDC on cell viability, SH-SY5Y cells were exposed to each LA at different concentrations (0.25–10 mM) and exposure times (20 min, 1 h, 4 h and 24 h). The results obtained using the resazurin assay are shown in Figure 2A. At the shortest exposure durations (20 min and 1 h), LDC showed higher cytotoxicity than ATC (IC50 ~7 mM for LDC and >10 mM for ATC). At 4 h, the cytotoxic profiles of the two LAs were similar, with IC50 values in the 5–6 mM range. At 24 h, the drug concentrations causing a 50% decrease in cell viability were 3.0 ± 0.2 mM and 3.6 mM ± 0.4 mM, for ATC and LDC, respectively, with no significant differences between them.

For both LAs, cell viability decreased in a time- and concentration-dependent manner, in agreement with the data previously reported for LDC in the same cell line [22,32]. The lower toxicity of ATC compared to LDC at short exposure times also agrees with previous studies [26,27]. Malet et al. reported LD50 values of 3.3 mM and 9.0 mM, upon 20 min incubation of SH-SY5Y cells with LDC and ATC, respectively [27]. At even shorter (5 min) exposure time, ATC produced no effect [26]. The LAs’ cytotoxicity and pharmacological effects are directly related to their physical–chemical properties, as only the neutral species (pKa LDC and ATC = 7.8) can partition in the cell membrane and reach the acting site on transmembrane proteins [7]. The neutral form of LDC is ~10 times more liposoluble than that of ATC (vide partition coefficients in Figure 1). Hence, it is likely that LDC inserts into the cell membrane more rapidly than ATC, producing a stronger effect at short exposure times.

Despite the short plasma half-lives of ATC (20 min) and LDC (90 min) [10], the local tissue exposure to LAs can reach up to 4 h (time of soft tissue anesthesia using mandibular block technique [11]). Hence, it is important that toxicity tests also include longer times. Considering the 24 h exposure time, the IC50 obtained here for LDC (3.6 mM) is very similar to that reported by Wang et al. in the same cell line [33]. Moreover, we observed no significant differences between ATC and LDC cytotoxicity at that time. Furthermore, 24 h LD50 values of 13.4 mM and 9.2 mM were previously reported for ATC and LDC, respectively, but in neuronal cells of a different lineage (SHEP) [23]. Overall, our results are in line with the general observation that the toxicity of ATC is lower or comparable to that of LDC, both in vitro, considering different cell lines [34,35], and in vivo [36].

Regarding alterations in cell morphology (Figure 2B), the 24 h IC10 concentration of both anesthetics (1.2 mM) caused few changes, with SH-SY5Y cells retaining the neuroblast-like morphology seen in controls [28]. However, at the IC50 concentrations, cells displayed a less extended, more rounded morphology, in agreement with previous reports [27,32]. This effect appeared to be more pronounced for LDC, probably due to its higher tendency to disturb the cell membrane compared to other LAs [37].

### 2.2. ^1^H NMR-Metabolomics

#### 2.2.1. Metabolic Profiling of Culture Medium

**Figure 3:**
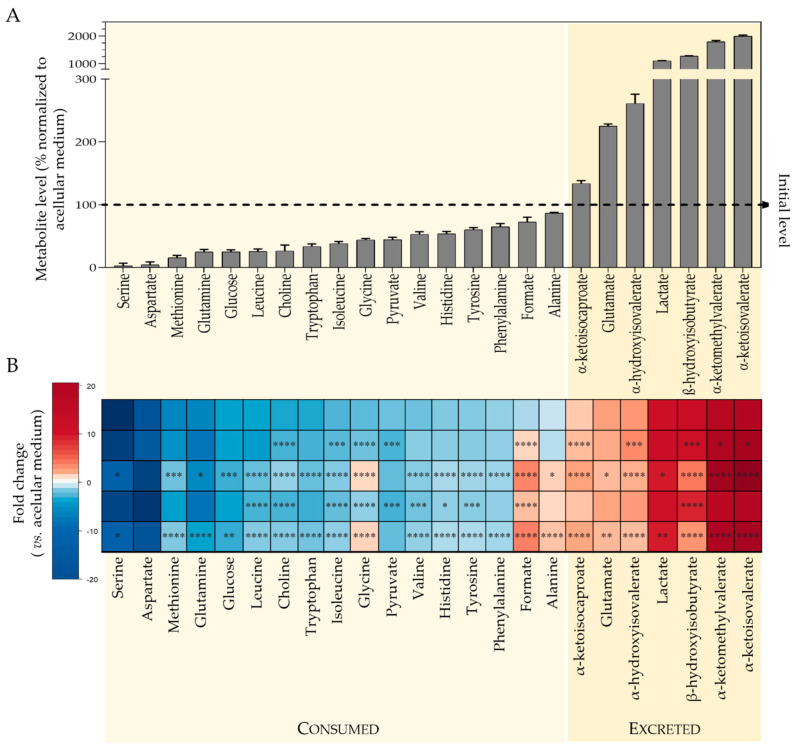
Results of metabolic profiling of culture medium. (**A**) Metabolite levels in the medium of control cells normalized to acellular medium: for each metabolite, the signal area in the medium resulting from incubating cells for 24 h was divided by the signal area in the acellular medium (set to 100%, dashed line); hence, metabolites with levels below 100% were consumed by cells, whereas metabolites with levels above 100% were excreted. (**B**) Heatmap colored according to fold change (FC) in cells-conditioned medium vs. acellular medium, showing the comparison for control cells and cells exposed for 24 h to IC10 or IC50 concentrations of articaine (ATC10 and ATC50, respectively) and to IC10 or IC50 concentrations of lidocaine (LDC10 and LDC50, respectively). Metabolites consumed show negative FC (blue scale), while metabolites excreted display positive FC (red scale). The color intensity reflects the magnitude of consumption/excretion. Statistical significance was assessed in respect to controls using the *t*-test (* *p* < 0.05; ** *p* < 0.01; *** *p* < 0.005; **** *p* < 0.001).

^1^H NMR analysis of culture media enabled the metabolic activity of SH-SY5Y cells to be readily assessed. Twenty-four medium components significantly changed their levels upon incubation with cells, reflecting metabolite consumption and excretion. Quantitative variations are presented in Figure 3A, which shows the % level of each metabolite, in the cells-conditioned medium, relatively to the acellular culture medium (representing 100%). Cells consumed considerable amounts of amino acids: over 95% of serine and aspartate, ~85% glutamine and methionine, ~75% leucine and tryptophan, together with varying amounts of other branched chain and aromatic amino acids, ~60% glycine and ~50% histidine. Other highly consumed substrates were glucose and choline (~75% consumption), pyruvate (56%) and formate (28%). On the other hand, metabolites secreted by cells included lactate (levels more than 10 times higher than in acellular medium), glutamate, branched chain keto acids (BCKA: α-ketoisocaproate or KIC, α-ketoisovalerate or KIV, α-ketomethylvalerate or KMV), β-hydroxyisobutyrate (HIB) and α-hydroxyisovalerate (HIV).

Exposure to ATC and LDC significantly affected the consumption and excretion patterns of several metabolites, as summarized in the heatmap shown in Figure 3B and depicted in more detail in Figure S1. Most effects were common to the two LAs, and, as expected, the IC50 concentration had a higher impact than IC10. The only exception was seen for pyruvate, the consumption of which increased significantly in cells treated with ATC or LDC at IC10, whereas it showed no difference to controls at IC50. Other than that, exposed cells showed decreased consumption of glucose, choline and most amino acids, except for aspartate (for which no differences were observed).

Regarding excreted metabolites, drug-exposed cells released higher amounts of BCKA than control cells, suggesting an impact in the metabolism of branched chain amino acids (BCAA: valine, leucine and isoleucine). Additionally, glycine and formate, which were consumed under control conditions, and alanine (unchanged by control cells) were secreted by treated cells in a concentration-dependent way. On the other hand, the secretion of lactate, glutamate, HIB and HIV decreased upon exposure to ATC or LDC.

#### 2.2.2. Metabolic Profiling of Cell Aqueous Extracts

**Figure 4:**
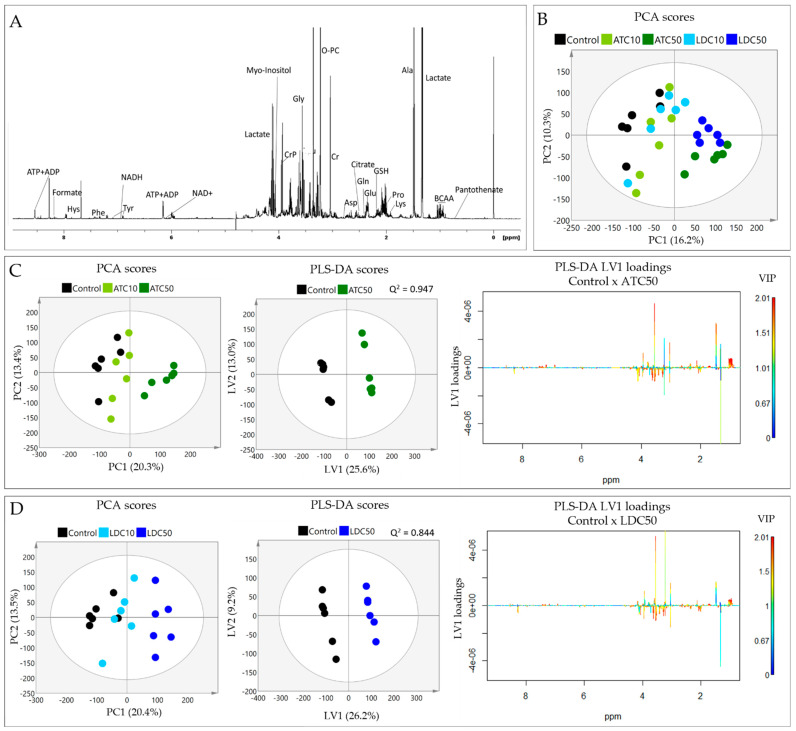
Results of metabolic profiling of cell aqueous extracts. (**A**) 1D ^1^H-NMR spectrum of a polar extract of SH-SY5Y control cells, with some assignments indicated (three-letter codes used for amino acids; ATP/ADP, adenosine tri/di-phosphate; BCAA, branched chain amino acids; NAD, nicotinamide adenine dinucleotide; PC, phosphocholine; PCr, phosphocreatine). (**B**) Principal Component Analysis (PCA) scores scatter plot of the five sample groups compared (control and exposed to IC10 and IC50 concentrations of ATC or LDC). (**C**) PCA and Partial Least Squares Discriminant Analysis (PLS-DA) of (**C**) control and ATC-exposed cells, (**D**) control and LDC-exposed cells: PCA scores scatter plot (left) showing the separation between control, IC10 and IC50; PLS-DA scores scatter plot (middle) showing the discrimination between control and IC50-exposed cells; PLS-DA LV1 loadings plot (right) colored according to variable importance to the projection (VIP), showing the spectral signals responsible for group discrimination.

^1^H NMR analysis of SH-SY5Y polar extracts enabled the detection of about 40 metabolites, including organic acids, nucleotides, amino acids and derivatives (Figure 4A and Supplementary Table S1). The impact of the two LAs on the intracellular metabolic composition was first assessed by multivariate analysis of spectral profiles. Principal Component Analysis (PCA) of the five sample groups showed a clear separation along PC1 (16% variance explained) between the groups exposed to high LA concentrations (ATC50 and LDC50) and those exposed to lower drug concentrations (ATC10 and LDC10), which clustered close to controls (Figure 4B). The separation between ATC and LDC, at either IC10 or IC50, was not clear in the PCA scores scatter plot. Hence, we proceeded to analyze each LA separately, in relation to untreated controls (Figure 4C,D, left). In both cases, the PCA scores of the IC10 samples were only slightly separated from controls, while IC50 samples were clearly apart, reflecting a dose-dependent impact on the metabolic profiles.

Partial Least Squares Discriminant Analysis (PLS-DA) further confirmed the robust discrimination of ATC50 and LDC50 groups from control cells, with high predictive powers (Q^2^ values > 0.8) being obtained by cross validation in both models (Figure 4C,D, middle). The respective LV1 loadings profiles, colored according to variable importance to the projection (VIP), revealed the main signals responsible for the observed group discrimination (Figure 4C,D, right). These included BCAA, alanine, creatine, glycine and aromatic amino acids (increased in LA-treated vs. control), together with lactate, proline, phosphocreatine and myo-inositol (decreased in LA-treated vs. control). Notably, the loadings signatures were very similar for ATC and LDC exposure, with subtle differences being noticed mainly in the importance of some variables.

**Figure 5:**
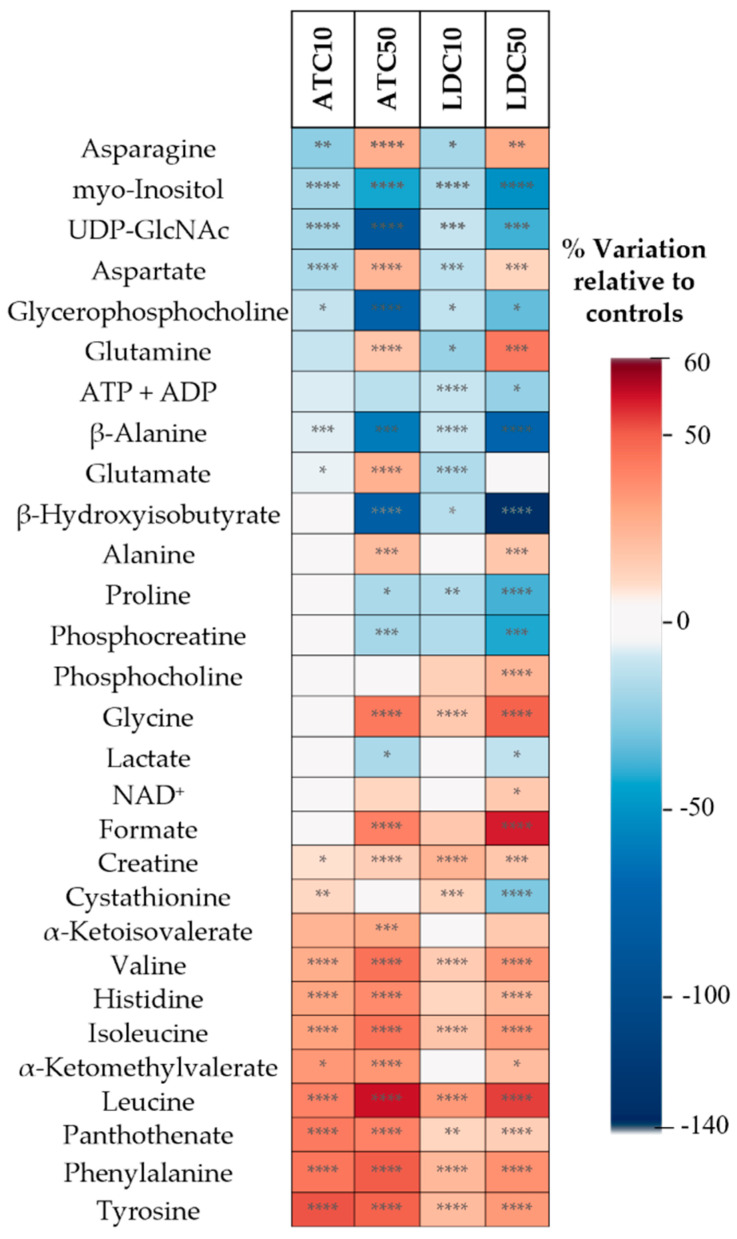
Heatmap summarizing the metabolic variations detected by NMR analysis of aqueous extracts, as assessed by spectral integration. The color scale represents the % variation in ATC- or LDC-exposed cells (at IC10 and IC50 concentrations) in relation to untreated controls. Hence, metabolites with lower levels in LA-exposed cells than in untreated controls show negative variations (blue color), while metabolites increased in exposed cells relative to controls display positive variations (red color). Statistical significance was assessed in respect to controls using the *t*-test (* *p* < 0.05; ** *p* < 0.01; *** *p* < 0.005; **** *p* < 0.001). ADP, adenosine diphosphate; ATP, adenosine triphosphate; NAD, nicotinamide adenine dinucleotide; UDP-GlcNAc, uridine diphosphate N-acetylglucosamine.

To assess the magnitude of the effects triggered by each LA, the relative variations in individual metabolites were assessed through spectral integration. Twenty-eight metabolites showed significant differences in exposed vs. control cells in at least one exposure condition (Figure 5 and Supplementary Table S2). As indicated by PCA, the cellular response was clearly dose-dependent, with the IC50 concentration producing statistically significant (*p* < 0.05) changes in a higher number of metabolites than IC10 (25 vs. 17 for ATC; 27 vs. 20 for LDC). Most metabolites varied in same direction at both concentrations, showing stronger variations at the IC50. However, there were a few exceptions to this observation, namely for asparagine, aspartate, glutamine and glutamate, which decreased after IC10 exposures and increased at the IC50 (glutamate showed a slightly different behavior, though, with LDC-treated cells displaying unaltered levels at the IC50). On the other hand, cystathionine increased at the IC10 of both ATC and LDC and showed no variation (ATC) or decrease (LDC) at the IC50 exposures.

In agreement with the similar loading signatures obtained by PLS-DA, most effects were common to ATC and LDC, although with varying magnitudes. Considering the IC50 concentration, both LAs induced decreases in the intracellular levels of myo-inositol, uridine diphosphate-N-acetylglucosamine (UDP-GlcNAc), glycerophosphocholine (GPC), ADP+ATP, β-alanine, β-hydroxyisobutyrate (HIB), proline, phosphocreatine and lactate. On the other hand, the levels of several amino acids (asparagine, aspartate, glutamine, alanine, glycine, BCAA, histidine, tyrosine and phenylalanine), NAD^+^, formate, creatine, two BCKA, and pantothenate were increased at the IC50 of both LAs. Apart from differences in magnitude, differential variations induced by the two LAs at the IC50 comprised glutamate (increased by ATC, not altered by LDC) and phosphocholine (not altered by ATC, increased by LDC).

#### 2.2.3. Metabolic Profiling of Cell Lipid Extracts

**Figure 6:**
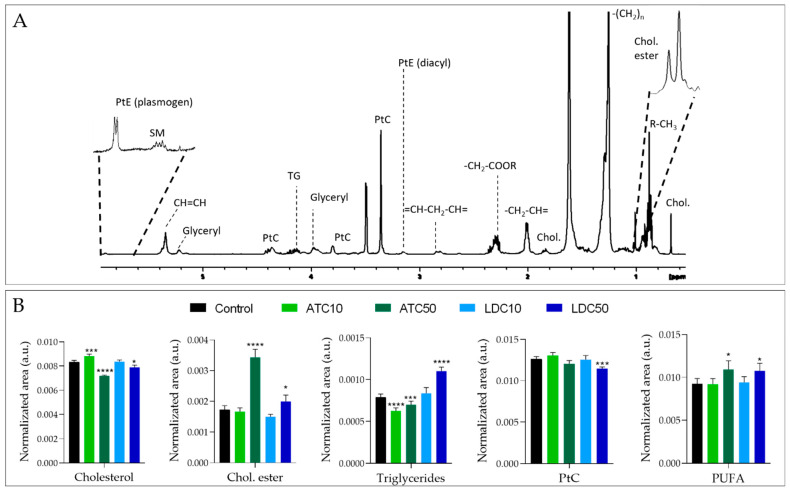
Results of metabolic profiling of cell lipid extracts (**A**) 1D ^1^H NMR spectrum of an apolar extract of SH-SY5Y control cells. (**B**) Relative levels of some lipid classes, obtained by spectral integration and normalization to total area, in control samples and in cells exposed to ATC or LDC at the IC10 or IC50 concentrations. Statistical significance was assessed in respect to controls using the *t*-test (* *p* < 0.05; *** *p* < 0.005; **** *p* < 0.001). Chol, cholesterol; Chol ester, cholesteryl esters; PtC, phosphatidylcholine; PtE, phosphatidylethanolamine; PUFA, polyunsaturated fatty acyl chains; SM, sphingomyelin; TG, triglycerides.

The ^1^H NMR spectrum of the cells’ lipid fraction shows signals from the fatty acyl chains and the glyceryl backbone of different lipid molecules, choline and ethanolamine headgroups in phospholipids, cholesterol and cholesterol esters (Figure 6A). The significant changes induced in lipid levels by ATC or LDC are shown in Figure 6B. Again, the magnitude of variations depended on the LA concentration. Considering the effects seen at the IC50 exposure (more pronounced), ATC induced a significant decrease in cholesterol, accompanied by a prominent increase in cholesteryl esters, together with decreases in triglycerides and phospholipids. As for LDC, similar variations were observed, except for triglycerides, which accumulated in LDC-treated cells. Finally, both LAs induced an increase in the amount of polyunsaturated fatty acids (PUFA).

## 3. Discussion

**Figure 7:**
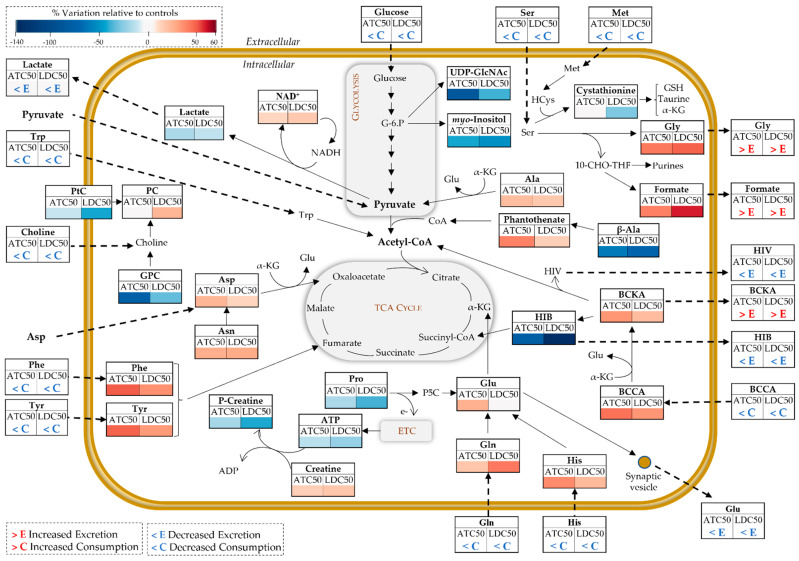
Schematic representation of metabolic reprogramming induced by ATC and LDC, at IC50 concentrations, in SH-SY5Y cells. Metabolite fluxes between extra- and intracellular compartments are indicated by dashed arrows. Intracellular variations are color-coded as in the heatmap in Figure 5. Three-letter codes used for amino acids; BCCA, branched chain amino acids (val, leu, ile); BCKA, branched chained keto acids (α-ketoisocaproate, α-ketoisovalerate, α-ketomethylvalerate); ETC, electron transport chain; GPC, glycerophosphocholine; HCys, homocysteine; HIB, β-hydroxyisobutyrate (HIB); HIV, α-hydroxyisovalerate; α-KG, α-ketoglutarate; NAD, nicotinamide adenine dinucleotide; PC, phosphocholine; PtC, phosphatidylcholine; P5C, pyrroline-5-carboxylate; UDP-GlcNAc, uridine diphosphate N-acetyl glucosamine.

The metabolic variations described in the previous sections reveal a pronounced impact of ATC and LDC on central metabolic pathways of neuronal cells, as schematically summarized in Figure 7 and described below. For the sake of clarity, this discussion concerns the effects seen at the higher LA concentration, unless otherwise specified.

In control conditions, SH-SY5Y cells consumed about 75% of the glucose present in the medium and secreted high amounts of lactate. Glucose is metabolized via glycolysis to pyruvate, which can then be converted to lactate or enter the tricarboxylic acid (TCA) cycle as acetyl-CoA. Compared to other brain cells, namely, astrocytes, neurons have been described as less glycolytic [38]. Still, they do take up glucose via the GLUT3 transporter [39] and have the capacity to export lactate [40]. Moreover, it should be noted that the neoplastic nature of SH-SY5Y cells is consistent with its lactate-producing glycolytic phenotype (Warburg effect), as previously reported [41].

When exposed to either ATC or LDC, SH-SY5Y cells decreased glucose consumption, lactate secretion and intracellular lactate levels, indicating lower glycolytic activity. This is in line with the results previously reported for LDC in different cell types, whereby this drug inhibited glycolysis by interfering with membrane hexose transporters [42], inducing a dose-dependent detachment of glycolytic enzymes (phosphofructokinase and aldolase) from the cytoskeleton of B16 melanoma cells [43], or causing oxidative damage to glyceraldehyde-3-phosphate dehydrogenase (GAPDH) and phosphoglycerate kinase (PGK1) [44]. The lower glucose uptake could also be linked to another change observed here—the significant dose-dependent decrease in UDP-GlcNAc. This metabolite, synthetized from glucose-6-phosphate through the hexosamine biosynthetic pathway, is the substrate for protein O-GlcNAcylation, a post-translational modification of critical importance to various neuronal functions [45].

In addition to glucose, SH-SY5Y cells consumed significant amounts of pyruvate and several amino acids, likely used to fuel the TCA cycle and mitochondrial oxidative phosphorylation (OXPHOS). Aspartate and serine were consumed nearly to exhaustion under control conditions. Aspartate is essential for transferring reducing equivalents from the cytosol to the mitochondria, through the malate-aspartate shuttle, which is very active in neurons [46]. Serine plays key roles in brain cells, including in (i) neurotransmission, as it is a precursor for glycine and D-serine (two regulators of the excitatory glutamatergic transmission), (ii) the biosynthesis of new cellular components, through its participation in one-carbon metabolism and (iii) antioxidant defenses, due to its involvement in the synthesis of glutathione [47]. As neuronal cells do not synthetize serine, it must be supplied by the extracellular medium. In the presence of ATC and LDC, both serine and methionine were less-consumed by SH-SY5Y cells. Together with the observed intracellular accumulation of glycine and formate and their release to the culture medium (contrarily to their consumption by control cells), these observations suggest downregulation of one-carbon metabolism by the LAs, possibly in relation to lower cell proliferation. Interestingly, the formate overflow linked to serine metabolism has been postulated to contribute to energy generation in cancer cells [48].

Glutamine was also actively consumed by cells, while glutamate was secreted. This matches the physiological situation where neurons take up the glutamine released by astrocytes and convert it into glutamate, which is then released during synaptic transmission and captured by astrocytes to produce glutamine and complete the cycle [49]. Moreover, apart from its role as a major neurotransmitter, glutamate is utilized as a substrate to support energy metabolism, through its conversion to the TCA cycle intermediate α-ketoglutarate (α-KG). Upon exposure to ATC and LDC at the IC50, glutamine consumption and glutamate secretion decreased, whereas their intracellular levels increased (or did not vary), possibly reflecting lower utilization. Overall, this suggests LA-induced disturbance of glutamate functions, both in energy metabolism and synaptic transmission.

The metabolic activity of control SH-SY5Y cells was further characterized by the consumption of BCAA and the secretion of BCKA and their derivatives. The catabolism of BCAA serves important functions in brain cells, as their transamination with α-KG enables the production of glutamate. Moreover, the BCKA resulting from those transamination reactions (KIC, KMV and KIV) can be oxidatively decarboxylated to produce acyl-CoA derivatives, which fuel the TCA cycle for energy production and/or lipid synthesis [50]. Indeed, Gondáš et al. elegantly demonstrated that SH-SY5Y cells catabolized leucine into acetyl-CoA to supply the TCA cycle [51]. In this work, we show that ATC and LDC have a major impact on neuronal cells’ BCAA catabolism. In LA-exposed cells, the uptake of BCAA from the culture medium was reduced, while their intracellular levels increased, together with intracellular accumulation and release of BCKA. On the other hand, the secretion of HIB and HIV (resulting from the transformation of BCKA) decreased. Altogether, these data strongly suggest that BCAA metabolism, especially the second irreversible step of BCKA decarboxylation, was affected by LA exposure. One of the consequences of such disturbance would be lower production of acetyl-CoA via this pathway. On the other hand, it appears that LA-exposed cells intensified the use of β-alanine to produce pantothenate (a component of coenzyme A), which could represent an alternative way to obtain acetyl-CoA.

Several other amino acids (phenylalanine, tyrosine, tryptophan, histidine) showed the same variation pattern upon ATC or LDC exposure, i.e., lower consumption and intracellular accumulation in LA-treated cells, again suggesting downregulation of TCA cycle anaplerotic fueling. Increased intracellular levels of amino acids in neuroblastoma cells have also been reported in response to other substances, such as the neurotoxic metabolite β-Methylamino-l-alanine (BMAA) [52] and the chemotherapy agent cisplatin [53]. In particular, cisplatin-resistant neuroblastoma cells showed heightened amino acid levels upon exposure to cisplatin, while cell survival and drug resistance decreased when the culture medium was deprived of essential amino acids. Such an important role of amino acids metabolism in the survival and energetic homeostasis of neuroblastoma cells has been corroborated by others, e.g., in a study showing that medium supplementation with L-serine (and other amino acids) abrogated ROS production and enhanced ATP production [54]. In this work, we hypothesize that LDC and ATC reduce the anaplerotic use of amino acids, affecting energy production. Indeed, the interference of LDC in mitochondrial metabolism has been reported in previous studies [22,30,31,44,55]. For instance, LDC induced oxidative modification of the TCA cycle enzyme aconitase and of multiple ATP synthase subunits in model yeast cells, causing a reduced ATP content [30,44], and disturbed the levels of TCA cycle intermediates in breast cancer cells [31]. This LA was also reported to suppress the mitochondrial ETC in SH-SY5Y cells, thereby attenuating the mitochondrial membrane potential and triggering excessive ROS production [22]. In the present study, we additionally observed a slight decrease in ADP+ATP content, corroborating the impairment of cellular energy production, likely through inhibition of both glycolysis and mitochondrial metabolism. Furthermore, the higher phosphocreatine to creatine conversion (indicated by their decreased and increased levels, respectively) suggests activation of this energy buffer system as a compensatory energy production mechanism, similarly to what was observed when SH-SY5Y cells were exposed to the toxic metabolite 1-Methyl-4-phenylpyridinium (MPP^+^) [56].

Interestingly, proline was the only amino acid with decreased intracellular levels in LA-treated cells. This nonessential proteinogenic amino acid has its own metabolic pathway, which is closely inter-related with glutamate and glutamine metabolism, and has key roles not only in protein structure, but also in cellular energetic and redox homeostasis [57]. Previous studies have reported that, under nutrient stress, cancer cells catabolize proline to generate ATP via the first step of its degradation by proline oxidase (PRODH) [58,59]. While converting proline to pyrroline-5-carboxylate (P5C), this enzyme passes electrons to FADH_2_, which can then enter the ETC to produce ATP [57]. Hence, it is possible that the hereby observed decrease in intracellular proline, triggered by ATC and LDC, reflects this mechanism, although this hypothesis requires confirmation.

Exposure to LA additionally triggered a dose-dependent decrease in the intracellular levels of myo-inositol, similarly to the variation observed in breast cancer cells treated with LDC [31]. This polyalcohol is a component of membrane phospholipids, mediates osmoregulation, and its phosphorylated derivatives participate in signal transduction pathways and other important cellular functions [60]. In the brain, myo-inositol can be transported from the blood or synthesized from glucose, while its efflux is an adaptive mechanism to osmotic stress [61]. For instance, SH-SY5Y cells in hypotonic medium were shown to release myo-inositol, an effect that was intensified upon stimulation of muscarinic cholinergic receptors [62]. In the present study, we may thus hypothesize that decreased myo-inositol levels in LA-exposed cells could arise from lower glucose uptake and/or an increased efflux, although its extracellular presence could not be verified due to signal overlap.

Changes in membrane-related metabolites were also detected upon exposure to ATC and LDC. A decreased consumption of choline was one of the effects observed. As SH-SY5Y cells are essentially noncholinergic (i.e., they produce little acetylcholine) [63], their choline uptake shall be mostly related to its incorporation into membrane phospholipids. Hence, its decreased import into cells likely reflects their lower proliferation. Moreover, both LAs decreased the intracellular levels of GPC, an effect also reported in MPP^+^-exposed SH-SY5Y cells, which the authors postulated arise from stimulation of GPC phosphodiesterase [56]. Interestingly, only LDC caused a dose-dependent increase in phosphocholine (PC), whereas its levels remained unaffected in ATC-exposed cells. This may possibly relate to another differential variation, consisting of the decrease in phosphatidylcholine (PtC), seen only in LDC-treated cells and likely reflecting a greater interference of this LA with the cell membrane.

Analysis of the cells’ lipid extracts further revealed changes in the amounts of neutral lipids. Cholesteryl esters increased, especially in ATC-exposed cells, while triglycerides increased in LDC-treated cells. Neutral lipids typically localize within lipid droplets in the cytosol of various cell types, including brain cells [64]. Besides their role in lipid storage, lipid droplets may serve other key functions, such as protection against lipotoxicity and redox imbalance. In neuronal cells, their build-up has been linked to situations of metabolic stress, aging and neurodegenerative diseases [65]. Hence, our newly reported hypothesis that ATC and LDC induce an increase in lipid droplets in neuronal cells, the composition of which depends on the specific LA employed, certainly deserves further investigation.

## 4. Materials and Methods

### 4.1. Chemicals

Dulbecco′s Modified Eagle′s Medium (DMEM)/Ham’s F-12 (DMEM:F12) with stable glutamine was purchase from Merck Biochrom (Berlin, Germany). Penicillin and streptomycin sulphate, fetal bovine serum (FBS), resazurin and lidocaine hydrochloride (LDC) were obtained from Sigma-Aldrich (St. Louis, MI, USA). Articaine hydrochloride (ATC) was donated by DFL Ind. Farm. (Rio de Janeiro, Brazil).

### 4.2. Cell Culture and Viability Assay

Undifferentiated SH-SY5Y human neuroblastoma cells (ATCC^®^ CRL-2266) were cultured in DMEM:F12 medium supplemented with 10% FBS and 1% antibiotic (100 IU/mLof penicillin and 100 μg/mL of streptomycin sulfate). Cells were grown in 75 cm^2^ bottles and incubated at 37 °C under a humidified atmosphere with 5% CO_2_ for 48 h, until reaching semiconfluency. All experiments were performed on cells between passages 4 to 8.

To assess cell viability, the resazurin test was used. This assay evaluates the ability of viable cells to reduce resazurin (blue color) to a fluorescent, pink-colored compound, resorufin. Briefly, the cells were plated in a 96-well plate 24 h before treatment, at a density of 4 × 10^4^ cells/mL. Cells were then incubated for 20 min, 1 h, 4 h or 24 h with several concentrations of ATC or LDC (0.25–10 mM). Each LA was solubilized in complete DMEM:F12 medium. Subsequently, the medium was removed, the wells were washed with 5 mM PBS and the cells were incubated with 10% resazurin for 4 h. At the end of this period, the absorbance was measured at 570 nm (λ1) and at 600 nm (λ2) (Multiskan™ FC Microplate Photometer, Thermofisher). The experiment was performed in triplicate and the differences between the two wavelengths (λ1–λ2) were calculated. The results were expressed in % of cell viability in relation to control and analyzed in GraphPad Prism^®^ 6.0.

### 4.3. ^1^H NMR Metabolomics

#### 4.3.1. Exposure of SH-SY5Y to the Local Anesthetics

After reaching semiconfluence, 10 mL of cells (1 × 10^6^ cells/mL) were plated on 90 mm diameter culture dishes and incubated for 24 h. ATC or LDC were then added to each plate at concentrations reducing cell viability by 10% (IC10; 1.2 mM) or 50% (IC50; 3.0 mM for ATC and 3.6 mM for LDC), as determined by the resazurin assay, and incubated for an additional 24 h. Afterward, the medium was collected and the dishes washed with cold PBS, immediately before the solvent extraction procedure described below. Unexposed cells were used as controls. Each assay comprised duplicates of each condition and three independent assays were performed, giving a total of 6 replicates per condition.

#### 4.3.2. Sample Extraction

The culture medium was collected from each plate (including cell-free medium incubated under the same conditions) in conical tubes and centrifuged at 1000× *g* for 10 min. Furthermore, 300 µL of the supernatant were collected for protein precipitation, as described by Kostidis et al. [66]. Briefly, 600 µL of 100% methanol (*v*/*v*) at −80 °C were added to the collected supernatant (ratio 1:3). The mixture was kept at −20 °C for 30 min and centrifuged at 13,000× *g* for 20 min. The supernatant was transferred to another tube and vacuum dried (SpeedVac, Eppendorf, Hamburg, Germany). For extracting cellular metabolites, a biphasic extraction protocol (methanol:chloroform:water; 1:1:0.7) was used as previously described [67]. The resulting polar extracts were dried under vacuum in a Speedvac concentrator, and the lipophilic extracts were dried under a nitrogen flow. All extracts were stored at −80 °C prior to NMR analysis.

#### 4.3.3. NMR Data Acquisition and Processing

For NMR analysis, dried aqueous and lipid extracts were dissolved, respectively, in 600 μL of deuterated phosphate buffer (PBS 100 mM, pH 7.4) containing 0.1 mM 3-(trimethylsilyl) propionic acid (TSP-*d_4_*), and 600 μL of deuterated chloroform containing 0.03% tetramethylsilane (TMS). After transferring 550 μL of each sample to 5 mm NMR tubes, analysis was performed on a Bruker Avance III HD 500 NMR spectrometer (University of Aveiro, Portuguese NMR Network) operating at 500.13 MHz for ^1^H observation, at 298 K. Standard 1D ^1^H spectra (pulse programs ‘noesypr1d’ and ‘zg’ for aqueous and lipid samples, respectively) were recorded with 32 k points, 7002.80 Hz spectral width, a 2 s relaxation delay and 512 scans. Spectral processing in TopSpin 4.0.3 (Bruker BioSpin, Rheinstetten, Germany) comprised cosine multiplication (ssb 2), zero-filling to 64 k data points, manual phasing, baseline correction and calibration to TSP-*d_4_*/TMS signals (δ 0 ppm). Two-dimensional NMR spectra, namely, ^1^H-^1^H TOCSY, *J*-resolved and ^1^H-^13^C HSQC spectra, were also recorded for selected samples to aid metabolite identification. Signal assignment was based on matching 1D and 2D spectral information to reference spectra available in Chenomx 9.0 (Edmonton, AB, Canada), BBIOREFCODE-2–0–0 (Bruker Biospin, Rheinstetten, Germany) and HMDB.

#### 4.3.4. Multivariate Analysis and Spectral Integration

Following spectra normalization by total area (excluding the suppressed water region, residual solvent signals and, in the case of medium and lipid extracts’ spectra, LA signals), data matrices (Table S3, Table S4 and Table S5) were uploaded into SIMCA-P 11.5 (Umetrics, Umeå, Sweden) and scaled to unit variance (UV) to give all variables equal variance. Principal Component Analysis (PCA) and Partial Least Squares-Discriminant Analysis (PLS-DA) were applied and the results were visualized through factorial co-ordinates (‘scores’) and factorial contributions (‘loadings’) colored according to variable importance to the projection (VIP). For PLS-DA models, Q^2^ and R^2^ values, respectively reflecting predictive capability and explained variance, obtained from sevenfold internal cross validation, were used to assess the robustness of group discrimination.

To provide a quantitative measurement of metabolic variations, spectral integration and total area normalization of selected signals (vide Supplementary Table S1) were carried out in Amix-Viewer 3.9.15 (Bruker Biospin, Rheinstetten, Germany). For each metabolite, the percentage of variation (or fold change) in exposed samples was calculated relative to respective controls, along with the effect size (ES) [68] and statistical significance (*p*-value). The variations with larger magnitude (|ES| > 0.8) were expressed in a heatmap. Loadings profiles and heatmap figures were generated using the R software version 4.1.3 (R Core Team (2020). R: A language and environment for statistical computing. R Foundation for Statistical Computing, Vienna, Austria. URL http://www.R-project.org/ accessed 23 May 2022).

### 4.4. Statistical Analysis

The results were shown as mean ± SD. The statistical significance of differences between experimental groups was determined in GraphPad Prism (GraphPad Software, Inc., La Jolla, CA, USA). When comparisons were made between several groups, one-way analysis of variance (ANOVA), with a Sidak multiple comparison test, was employed. Otherwise, the unpaired two-tailed Student’s *t*-test for small sample sizes was used. Statistical differences were indicated as * *p* < 0.05, ** *p* < 0.01, *** *p* < 0.005, **** *p* < 0.001.

## 5. Conclusions

The two local anesthetics (LAs) hereby studied, ATC and LDC, showed comparable cytotoxicity to SH-SY5Y neuroblastoma cells at 4 and 24 h exposures, whereas ATC was less cytotoxic than LDC at shorter times (20 min and 1h). Their general impact on the cells’ metabolism was also very similar, although a few differences could be noted, mainly related to membrane disturbance and lipid metabolism. The metabolic variations elicited by the two LAs suggested (i) downregulation of glycolysis and of glucose-dependent pathways (e.g., one-carbon metabolism and hexosamine biosynthetic pathway), (ii) disturbance of branched chain amino acids (BCAA) catabolism, (iii) downregulation of TCA cycle anaplerotic fueling, together with activation of alternative energy-producing pathways, (iv) interference with choline metabolism and (v) lipid droplet build-up. Altogether, this work has provided a great amount of new information on key aspects of cell physiology, namely regarding bioenergetic balance and lipid metabolism, which helps to improve the current understanding of the molecular mechanisms involved in LAs’ neurotoxicity.

## Data Availability

The data that supports the findings of this study are available within the article and its supplementary materials.

## Supplementary Materials

The following supporting information can be downloaded at: https://www.mdpi.com/article/10.3390/metabo12070581/s1, Figure S1: Relative levels of extracellular metabolites in cells-conditioned medium, normalized to the initial levels in the acellular medium, Table S1: Metabolites identified in the cells aqueous extracts based on 1D and 2D NMR spectral data; Table S2: Metabolite variations in the aqueous extracts of ATC- and LDC-treated cells in relation to controls; Table S3: NMR data matrix corresponding to aqueous extracts; Table S4: NMR data matrix corresponding to lipid extracts; Table S5: NMR data matrix corresponding to culture media.

## References

[1] Seward G.R. (1981). Handbook of Local Anesthesia.

[2] Katyal V. (2010). The Efficacy and Safety of Articaine versus Lignocaine in Dental Treatments: A Meta-Analysis. J. Dent..

[3] Zhang A., Tang H., Liu S., Ma C., Ma S., Zhao H. (2019). Anesthetic Efficiency of Articaine Versus Lidocaine in the Extraction of Lower Third Molars: A Meta-Analysis and Systematic Review. J. Oral Maxillofac. Surg..

[4] Kakroudi S.H.A., Mehta S., Millar B.J. (2015). Articaine Hydrochloride: Is It the Solution?. Dent. Update.

[5] Bartlett G., Mansoor J. (2016). Articaine Buccal Infiltration vs Lidocaine Inferior Dental Block—A Review of the Literature. BDJ.

[6] Malamed S.F. (2009). Sedation-E-Book: A Guide to Patient Management.

[7] de Araújo D.R., Ribeiro L.N.D.M., de Paula E. (2019). Lipid-Based Carriers for the Delivery of Local Anesthetics. Expert Opin. Drug Deliv..

[8] Prates É.T., Rodrigues da Silva G.H., Souza T.F., Skaf M.S., Pickholz M., de Paula E. (2020). Articaine Interaction with Phospholipid Bilayers. J. Mol. Struct..

[9] Fraceto L.F., Spisni A., Schreier S., de Paula E. (2005). Differential Effects of Uncharged Aminoamide Local Anesthetics on Phospholipid Bilayers, as Monitored by 1H-NMR Measurements. Biophys. Chem..

[10] Onal O., Saltali A.O., Apiliogullari S. (2016). Comments on “Local Anesthetic Systemic Toxicity”. Aesthetic Surg. J..

[11] Malamed S.F., Gagnon S., Leblanc D. (2001). Articaine Hydrochloride: A Study of the Safety of a New Amide Local Anesthetic. J. Am. Dent. Assoc..

[12] Garlsto G.A., Gaffen A.S., Lawrence H.P., Tenenbaum H.C., Haas D.A. (2010). Occurrence of Paresthesia after Dental Local Anesthetic Administration in the United States. J. Am. Dent. Assoc..

[13] Kingon A., Sambrook P., Goss A. (2011). Higher Concentration Local Anaesthetics Causing Prolonged Anaesthesia. Do They? A Literature Review and Case Reports. Aust. Dent. J..

[14] Hillerup S., Jensen R. (2006). Nerve Injury Caused by Mandibular Block Analgesia. Int. J. Oral Maxillofac. Surg..

[15] Ramadurai N., Gurunathan D., Samuel A.V., Subramanian E., Rodrigues S.J.L. (2019). Effectiveness of 2% Articaine as an Anesthetic Agent in Children: Randomized Controlled Trial. Clin. Oral Investig..

[16] Hopman A.J.G., Baart J.A., Brand H.S. (2017). Articaine and Neurotoxicity—A Review. Br. Dent. J..

[17] Stirrup P., Crean S. (2019). Does Articaine, Rather than Lidocaine, Increase the Risk of Nerve Damage When Administered for Inferior Alveolar Nerve Blocks in Patients Undergoing Local Anaesthesia for Dental Treatment? A Mini Systematic Review of the Literature. Br. Dent. J..

[18] Malamed S. Articaine 30 Years Later. https://www.oralhealthgroup.com/features/1003919408/.

[19] Verlinde M., Hollmann M.W., Stevens M.F., Hermanns H., Werdehausen R., Lirk P. (2016). Local Anesthetic-Induced Neurotoxicity. Int. J. Mol. Sci..

[20] Kim E.-J., Kim H.Y., Ahn J.-H. (2020). Neurotoxicity of Local Anesthetics in Dentistry. J. Dent. Anesth. Pain Med..

[21] Johnson M.E., Uhl C.B., Spittler K.-H., Wang H., Gores G.J. (2004). Mitochondrial Injury and Caspase Activation by the Local Anesthetic Lidocaine. Anesthesiology.

[22] Okamoto A., Tanaka M., Sumi C., Oku K., Kusunoki M., Nishi K., Matsuo Y., Takenaga K., Shingu K., Hirota K. (2016). The Antioxidant N-Acetyl Cysteine Suppresses Lidocaine-Induced Intracellular Reactive Oxygen Species Production and Cell Death in Neuronal SH-SY5Y Cells. BMC Anesthesiol..

[23] Werdehausen R., Fazeli S., Braun S., Hermanns H., Essmann F., Hollmann M.W., Bauer I., Stevens M.F. (2009). Apoptosis Induction by Different Local Anaesthetics in a Neuroblastoma Cell Line. Br. J. Anaesth..

[24] Okamoto A., Sumi C., Tanaka H., Kusunoki M., Iwai T., Nishi K., Matsuo Y., Harada H., Takenaga K., Bono H. (2017). HIF-1-Mediated Suppression of Mitochondria Electron Transport Chain Function Confers Resistance to Lidocaine-Induced Cell Death. Sci. Rep..

[25] Haller I., Hausott B., Tomaselli B., Keller C., Klimaschewski L., Gerner P., Lirk P. (2006). Neurotoxicity of Lidocaine Involves Specific Activation of the P38 Mitogen-Activated Protein Kinase, but not Extracellular Signal-Regulated or c-Jun N-Terminal Kinases, and Is Mediated by Arachidonic Acid Metabolites. Anesthesiology.

[26] Albalawi F., Lim J.C., DiRenzo K.V., Hersh E.V., Mitchell C.H. (2018). Effects of Lidocaine and Articaine on Neuronal Survival and Recovery. Anesth. Prog..

[27] Malet A., Faure M.O., Deletage N., Pereira B., Haas J., Lambert G. (2015). The Comparative Cytotoxic Effects of Different Local Anesthetics on a Human Neuroblastoma Cell Line. Anesth. Analg..

[28] Kovalevich J., Langford D. (2013). Considerations for the Use of SH-SY5Y Neuroblastoma Cells in Neurobiology. Methods Mol. Biol..

[29] Schultz L., Zurich M.G., Culot M., da Costa A., Landry C., Bellwon P., Kristl T., Hörmann K., Ruzek S., Aiche S. (2015). Evaluation of Drug-Induced Neurotoxicity Based on Metabolomics, Proteomics and Electrical Activity Measurements in Complementary CNS in vitro Models. Toxicol Vitr..

[30] Boone C.H.T., Grove R.A., Adamcova D., Seravalli J., Adamec J. (2017). Oxidative Stress, Metabolomics Profiling, and Mechanism of Local Anesthetic Induced Cell Death in Yeast. Redox Biol..

[31] Chamaraux-Tran T.N., Muller M., Pottecher J., Diemunsch P.A., Tomasetto C., Namer I.J., Dali-Youcef N. (2022). Metabolomic Impact of Lidocaine on a Triple Negative Breast Cancer Cell Line. Front. Pharmacol..

[32] Gong Q., Wen X., Li H., He J., Wang Y., Wu H., Wang H., Wang X. (2018). Up-Regulation of Cav3.1 Expression in SH-SY5Y Cells Induced by Lidocaine Hydrochloride. Artif. Cells Nanomed. Biotechnol..

[33] Wang Z., Liu Q., Lu J., Cao J., Wang X.Y., Chen Y. (2020). Lidocaine Promotes Autophagy of SH-SY5Y Cells through Inhibiting PI3K/AKT/MTOR Pathway by Upregulating MiR-145. Toxicol. Res..

[34] Werdehausen R., Braun S., Fazeli S., Hermanns H., Hollmann M.W., Bauer I., Stevens M.F. (2012). Lipophilicity but not Stereospecificity Is a Major Determinant of Local Anaesthetic-Induced Cytotoxicity in Human T-Lymphoma Cells. Eur. J. Anaesthesiol..

[35] Wu T., Smith J., Nie H., Wang Z., Erwin P.J., van Wijnen A.J., Qu W. (2018). Cytotoxicity of Local Anesthetics in Mesenchymal Stem Cells. Am. J. Phys. Med. Rehabil..

[36] Baroni D.B., Franz-Montan M., Cogo K., Berto L.A., Volpato M.C., Novaes P.D., Groppo F.C. (2012). Effect of Articaine on Mental Nerve Anterior Portion: Histological Analysis in Rats. Acta Odontol. Scand..

[37] de Paula E., Schreier S. (1995). Use of a Novel Method for Determination of Partition Coefficients to Compare the Effect of Local Anesthetics on Membrane Structure. Biochim. Biophys. Acta.

[38] Jha M.K., Morrison B.M. (2018). Glia-Neuron Energy Metabolism in Health and Diseases: New Insights into the Role of Nervous System Metabolic Transporters. Exp. Neurol..

[39] Simpson I.A., Dwyer D., Malide D., Moley K.H., Travis A., Vannucci S.J. (2008). The Facilitative Glucose Transporter GLUT3: 20 Years of Distinction. Am. J. Physiol. Endocrinol. Metab..

[40] Yellen G. (2018). Fueling Thought: Management of Glycolysis and Oxidative Phosphorylation in Neuronal Metabolism. J. Cell Biol..

[41] Alherz M., Lee D., Alshangiti A., Roddy D., O’Keeffe G., White R., Barry D. (2021). The Growth Response to Beta-Hydroxybutyrate in SH-SY5Y Neuroblastoma Cells Is Suppressed by Glucose and Pyruvate Supplementation. Neurochem. Res..

[42] Uesono Y., Toh-e A., Kikuchi Y., Araki T., Hachiya T., Watanabe C., Noguchi K., Terashima I. (2016). Local Anesthetics and Antipsychotic Phenothiazines Interact Nonspecifically with Membranes and Inhibit Hexose Transporters in Yeast. Genetics.

[43] Schwartz D., Beitner R. (2000). Detachment of the Glycolytic Enzymes, Phosphofructokinase and Aldolase, from Cytoskeleton of Melanoma Cells, Induced by Local Anesthetics. Mol. Genet. Metab..

[44] Boone C.H.T., Grove R.A., Adamcova D., Braga C.P., Adamec J. (2016). Revealing Oxidative Damage to Enzymes of Carbohydrate Metabolism in Yeast: An Integration of 2D DIGE, Quantitative Proteomics, and Bioinformatics. Proteomics.

[45] Lee B.E., Suh P.G., Kim J.I. (2021). O-GlcNAcylation in Health and Neurodegenerative Diseases. Exp. Mol. Med..

[46] McKenna M.C., Waagepetersen H.S., Schousboe A., Sonnewald U. (2006). Neuronal and Astrocytic Shuttle Mechanisms for Cytosolic-Mitochondrial Transfer of Reducing Equivalents: Current Evidence and Pharmacological Tools. Biochem. Pharmacol..

[47] Maugard M., Vigneron P.A., Bolaños J.P., Bonvento G. (2020). L-Serine Links Metabolism with Neurotransmission. Prog. Neurobiol..

[48] Meiser J., Tumanov S., Maddocks O., Labuschagne C.F., Athineos D., van den Broek N., Mackay G.M., Gottlieb E., Blyth K., Vousden K. (2016). Serine One-Carbon Catabolism with Formate Overflow. Sci. Adv..

[49] Andersen J.V., Markussen K.H., Jakobsen E., Schousboe A., Waagepetersen H.S., Rosenberg P.A., Aldana B.I. (2021). Glutamate Metabolism and Recycling at the Excitatory Synapse in Health and Neurodegeneration. Neuropharmacology.

[50] Sperringer J.E., Addington A., Hutson S.M. (2017). Branched-Chain Amino Acids and Brain Metabolism. Neurochem. Res..

[51] Gondáš E., Král’Ová Trančíková A., Baranovičová E., Šofranko J., Hatok J., Kowtharapu B.S., Galanda T., Dobrota D., Kubatka P., Busselberg D. (2022). Expression of 3-Methylcrotonyl-CoA Carboxylase in Brain Tumors and Capability to Catabolize Leucine by Human Neural Cancer Cells. Cancers.

[52] Engskog M.K.R., Ersson L., Haglöf J., Arvidsson T., Pettersson C., Brittebo E. (2017). β-N-Methylamino-l-Alanine (BMAA) Perturbs Alanine, Aspartate and Glutamate Metabolism Pathways in Human Neuroblastoma Cells as Determined by Metabolic Profiling. Amino Acids.

[53] Gunda V., Pathania A.S., Chava S., Prathipati P., Chaturvedi N.K., Coulter D.W., Pandey M.K., Durden D.L., Challagundla K.B. (2020). Amino Acids Regulate Cisplatin Insensitivity in Neuroblastoma. Cancers.

[54] Delic V., Griffin J.W.D., Zivkovic S., Zhang Y., Phan T.A., Gong H., Chaput D., Reynes C., Dinh V.B., Cruz J. (2017). Individual Amino Acid Supplementation Can Improve Energy Metabolism and Decrease ROS Production in Neuronal Cells Overexpressing Alpha-Synuclein. NeuroMol. Med..

[55] la Monaca E., Fodale V. (2012). Effects of Anesthetics on Mitochondrial Signaling and Function. Curr. Drug Saf..

[56] Amo T., Oji Y., Saiki S., Hattori N. (2019). Metabolomic Analysis Revealed Mitochondrial Dysfunction and Aberrant Choline Metabolism in MPP+-Exposed SH-SY5Y Cells. Biochem. Biophys. Res. Commun..

[57] Vettore L.A., Westbrook R.L., Tennant D.A. (2021). Proline Metabolism and Redox; Maintaining a Balance in Health and Disease. Amino Acids.

[58] Pandhare J., Donald S.P., Cooper S.K., Phang J.M. (2009). Regulation and Function of Proline Oxidase under Nutrient Stress. J. Cell. Biochem..

[59] Liu W., Glunde K., Bhujwalla Z.M., Raman V., Sharma A., Phang J.M. (2012). Proline Oxidase Promotes Tumor Cell Survival in Hypoxic Tumor Microenvironments. Cancer Res..

[60] Chhetri D.R. (2019). Myo-Inositol and Its Derivatives: Their Emerging Role in the Treatment of Human Diseases. Front. Pharmacol..

[61] Fisher S.K., Novak J.E., Agranoff B.W. (2002). Inositol and Higher Inositol Phosphates in Neural Tissues: Homeostasis, Metabolism and Functional Significance. J. Neurochem..

[62] Loveday D., Heacock A.M., Fisher S.K. (2003). Activation of Muscarinic Cholinergic Receptors Enhances the Volume-Sensitive Efflux of Myo-Inositol from SH-SY5Y Neuroblastoma Cells. J. Neurochem..

[63] Yamada T., Inazu M., Tajima H., Matsumiya T. (2011). Functional Expression of Choline Transporter-like Protein 1 (CTL1) in Human Neuroblastoma Cells and Its Link to Acetylcholine Synthesis. Neurochem. Int..

[64] Ralhan I., Chang C.L., Lippincott-Schwartz J., Ioannou M.S. (2021). Lipid Droplets in the Nervous System. J. Cell Biol..

[65] Islimye E., Girard V., Gould A.P. (2022). Functions of Stress-Induced Lipid Droplets in the Nervous System. Front. Cell Dev. Biol..

[66] Kostidis S., Addie R.D., Morreau H., Mayboroda O.A., Giera M. (2017). Quantitative NMR Analysis of Intra- and Extracellular Metabolism of Mammalian Cells: A Tutorial. Anal. Chim. Acta.

[67] Carrola J., Bastos V., Jarak I., Oliveira-Silva R., Malheiro E., Daniel-da-Silva A.L., Oliveira H., Santos C., Gil A.M., Duarte I.F. (2016). Metabolomics of Silver Nanoparticles Toxicity in HaCaT Cells: Structure–Activity Relationships and Role of Ionic Silver and Oxidative Stress. Nanotoxicology.

[68] Berben L., Sereika S.M., Engberg S. (2012). Effect Size Estimation: Methods and Examples. Int. J. Nurs. Stud..

