# Supplementary material for: Comparative Metabolomics Study of the Impact of Articaine and Lidocaine on the Metabolism of SH-SY5Y Neuronal Cells
Source: Metabolites. 2022 Jun 23;12(7):581. doi: 10.3390/metabo12070581 (PMC9323911; doi:10.3390/metabo12070581)
Supplement: Supplementary file 1 [file metabolites-12-00581-s001.zip › metabolites-1776928-supplementary.pdf]

Supplementary Information

Comparative metabolomics study of the impact of articaine and lidocaine on the metabolism of SH-SY5Y neuronal cells

Gustavo H. Rodrigues da Silva[1,2], Luís F. Mendes[1], Fabíola V. de Carvalho[2], Eneida de Paula[2], Iola F. Duarte[1*]

[1] CICECO—Aveiro Institute of Materials, Department of Chemistry, University of Aveiro, Aveiro, Portugal;
[2] Department of Structural and Functional Biology, Institute of Biology, University of Campinas (UNICAMP), Campinas, SP, Brazil.

*Correspondence: ioladuarte@ua.pt

Figure S1. Relative levels of extracellular metabolites in cells-conditioned medium, normalized to the initial levels in the acellular medium (100%). The graphs are grouped according to the differences detected in the medium of ATC- and/or LDC-treated cells compared to that of untreated controls. Statistical significance calculated in respect to controls (* p<0.05; **p<0.01; ***p<0.005; ****p<0.001).

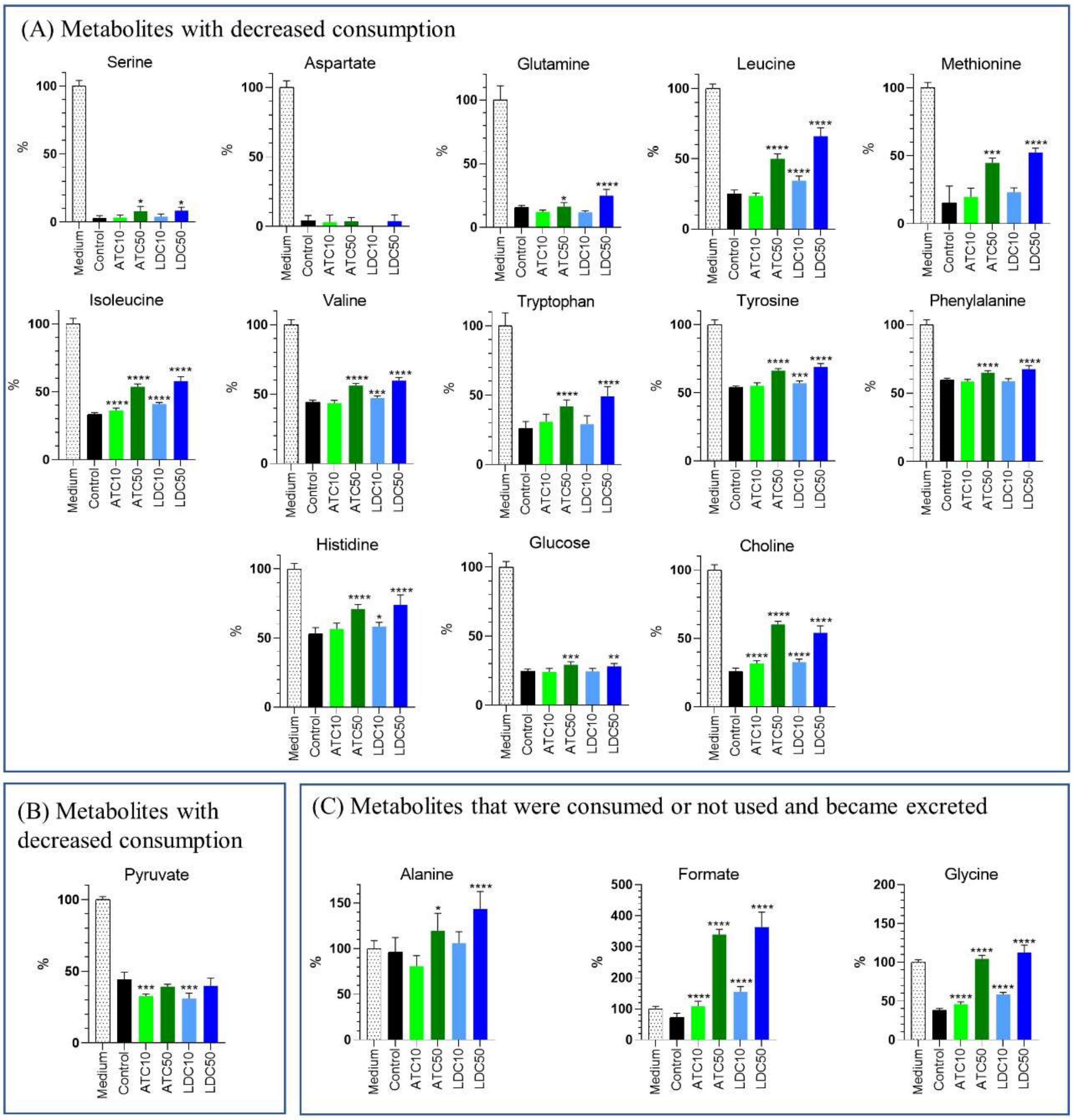

Figure S1 (cont.)

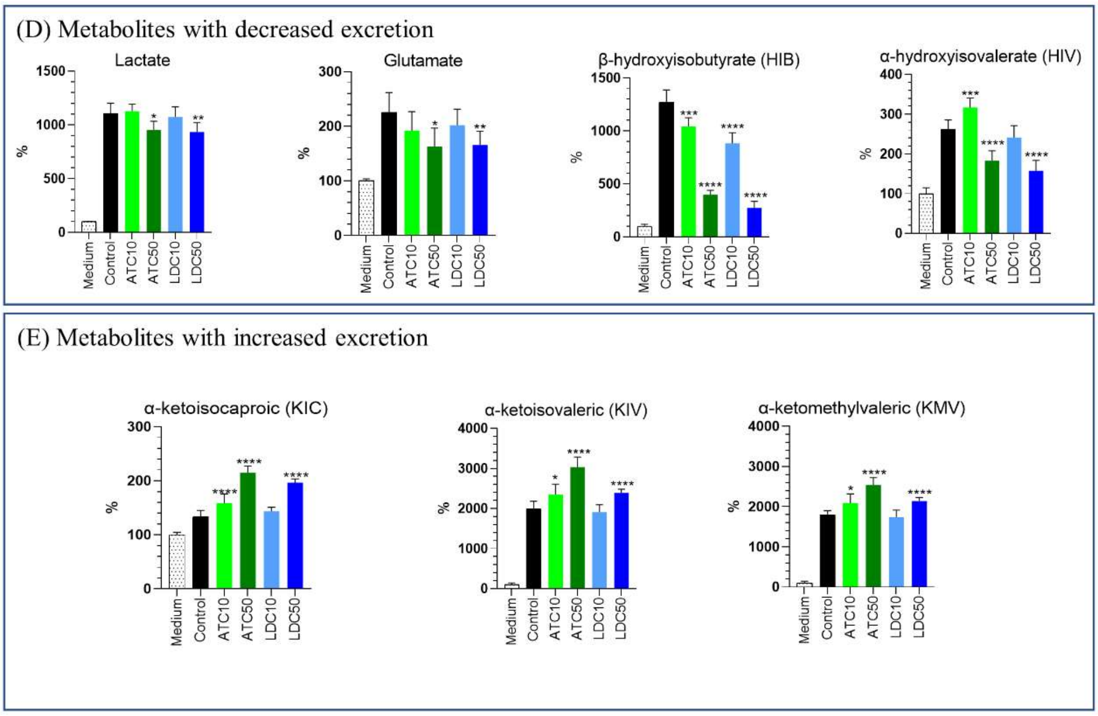

Table S1. Metabolites identified in the SH-SY5Y cells' aqueous extracts based on 1D and 2D NMR spectral data. The chemical shifts of signals selected for integration are in bold.

No.	Compound	δ ^{1}H in ppm (multiplicity)
**1**	Acetate	1.92 (s)
**2**	N-Acetylaspartate	**2.03** (s); 2.50 (dd); 2.69 (dd); 4.41 (dd)
**3**	ADP + ATP	4.23 (m); 4.39 (m); 4.59 (m); 4.85 (m); 6.16 (d); **8.28** (s); 8.53 (s)
**4**	Alanine	**1.49** (d); 3.78 (q)
**5**	β-Alanine	**2.57** (t); 3.19 (t)
**6**	Arginine	1.70 (m); 1.92 (m); 3.26 (t); 3.78 (t)
**7**	Asparagine	**2.88** (dd); 2.94 (dd), 3.99 (dd)
**8**	Aspartate	2.68 (dd); **2.81** (dd); 3.89 (dd)
**9**	Carnitine	2.44 (m); 3.23 (s); 3.43 (m); 4.57 (m)
**10**	Choline	**3.21** (s); 3.53 (m); 4.07 (m)
**11**	Citrate	2.54 (d); 2.66 (d)
**12**	Creatine	**3.04** (s); 3.94 (s)
**13**	Cystathionine	2.17 (m); **2.74** (m); 3.14 (m); 3.87 (m); 3.97 (m)
**14**	Formate	**8.46** (s)
**15**	Fumarate	**6.52** (s)
**16**	Glucose	3.25 (dd); 3.42 (m); 3.48 (m); 3.55 (dd); 3.74 (m); 3.85 (m); 3.90 (dd); 4.66 (d); **5.25** (d)
**17**	Glutamate	2.08 (m); **2.13** (m); 2.35 (m); 3.77 (dd)
**18**	Glutamine	2.15 (m); **2.46** (m); 3.77 (t)
**19**	Glycerophosphocholine	**3.24** (s); 3.68 (m); 3.91 (m); 4.32 (m)
**20**	Glycine	**3.57** (s)
**21**	GSH	2.17 (m); 2.55 (m); 2.95 (m); 3.80 (m); **4.57** (m)
**22**	GSSG	2.17 (m); 2.55 (m); 2.98 (m); 3.30 (m); 4.76 (m)
**23**	Histidine	3.23 (dd); 3.31 (dd); 4.01 (dd); **7.15** (s); 8.02 (s)
**24**	β-Hydroxyisobutyrate	**1.07** (d); 2.47 (m); 3.52 (dd); 3.69 (dd)
**25**	*myo*-Inositol	3.30 (t); 3.55 (dd); 3.63 (t); **4.08** (t)
**26**	Isoleucine	**0.94** (t); 1.01 (d); 1.26 (m); 1.48 (m); 1.98 (m); 3.67 (d)
**27**	α-Ketoisovalerate	**1.12** (d); 3.01 (m)
**28**	α-Ketomethylvalerate	0.90 (t); **1.10** (d); 1.48 (m); 1.74 (m); 2.94 (m)
**29**	Lactate	1.33 (d); **4.12** (q)
**30**	Leucine	**0.96** (t); 1.771 (m); 3.78 (m)
**31**	Lysine	1.48 (m); 1.73 (m); 1.91 (m); **3.03** (t); 3.79 (t)
**32**	NAD^+	6.04 (d); 6.10 (d); 8.18 (s); 8.20 (m); **8.42** (s); 8.84 (d); 9.15 (d); 9.35 (s)
**33**	Pantothenate	**0.90** (s); 0.93 (s); 2.44 (t); 3.40 (d); 3.44 (q); 3.52 (d); 3.99 (s)
**34**	Phenylalanine	3.13 (m); 3.28 (dd); 4.01 (m); **7.33** (d); 7.38 (d); 7.43 (t)
**35**	Phosphocholine	**3.23** (s) ; 3.60 (m) ; 4.16 (m)
**36**	Phosphocreatine	**3.05** (s); 3.96 (s)
**37**	Phosphoethanolamine	3.22 (t); 4.01 (m)
**38**	Proline	**2.00** (m); 2.08 (m); 2.35 (m); 3.36 (t); 3.41 (t); 4.14 (t)
**39**	Pyroglutamate	2.04 (m); 2.41 (m); 2.50 (m); 4.18 (dd)
**40**	Taurine	3.28 (t); 3.43 (t)
**41**	Threonine	1.33 (d); 3.61 (d); 4.25 (m)
**42**	Tyrosine	3.07 (m); 3.20 (m); 3.94 (m); **6.91** (d); 7.20 (d)
**43**	UDP-GlcNAc	**5.52** (dd); 5.95 (d); 5.99 (d); 7.95 (d)
**44**	Valine	0.99 (d); **1.03** (d); 2.28 (m); 3.62 (d)

Multiplicity: s, singlet; d, doublet; dd, doublet of doublets; dt, doublet of triplets; m, multiplet; q, quartet; t, triplet. ADP/ATP, adenosine di/triphosphate; GSH, reduced glutathione; GSSG, oxidized glutathione; NAD+, nicotinamide adenine dinucleotide; UDP-GlcNAc, uridine diphosphate-N-acetyl-glucosamine.

Table S2. Metabolite variations in the aqueous extracts of ATC- and LDC-treated cells in relation to controls. The values of % variation presented are those used to color-code the heatmap shown in Figure 4 of the manuscript.

	Variation in relation to control (%)				±				t-test (p-value)				Effect Size			
**Metabolites**	**ATC10**	**ATC50**	**LDC10**	**LDC50**	**ATC10**	**ATC50**	**LDC10**	**LDC50**	**ATC10**	**ATC50**	**LDC10**	**LDC50**	**ATC10**	**ATC50**	**LDC10**	**LDC50**
Asparagine	-24.6	26.5	-18.9	27.8	7.8	6.7	8.6	9.6	0.0050	0.0007	0.0367	0.0069	-1.9	2.6	-1.3	2.0
myo-Inositol	-19.0	-42.6	-16.9	-50.6	1.8	1.6	2.2	1.8	0.0000	0.0000	0.0000	0.0000	-6.2	-11.8	-4.5	-11.9
UDP-GlcNAc	-18.7	-85.9	-10.8	-38.7	3.5	2.7	3.0	6.6	0.0002	0.0000	0.0036	0.0014	-3.1	-11.4	-2.0	-2.8
Aspartate	-17.2	23.7	-12.9	12.1	6.1	3.8	5.9	4.8	0.0119	0.0005	0.0416	0.0220	-1.6	3.9	-1.2	1.5
Glycerophosphocholine	-11.3	-75.7	-11.7	-33.4	7.2	7.7	5.4	6.4	0.1305	0.0000	0.0472	0.0011	-0.9	-3.9	-1.2	-2.4
Glutamine	-10.8	17.8	-22.0	43.0	7.3	13.2	5.1	18.9	0.1587	0.1568	0.0007	0.0215	-0.8	0.9	-2.6	1.7
ATP + ADP	-8.0	-14.0	-10.4	-23.0	2.1	3.0	2.2	2.6	0.0031	0.0014	0.0006	0.0000	-2.1	-2.4	-2.7	-4.4
beta-Alanine	-7.4	-60.7	-10.0	-72.1	2.4	1.9	1.4	2.1	0.0178	0.0000	0.0000	0.0000	-1.7	-13.8	-4.1	-14.0
Glutamate	-6.3	25.8	-16.3	0.0	2.3	7.2	4.0	3.5	0.0178	0.0051	0.0029	0.7153	-1.5	2.4	-2.4	0.0
Isobutyrate	0.0	-78.2	-14.4	-131.7	4.4	5.2	5.7	5.3	0.4303	0.0000	0.0222	0.0000	0.0	-5.7	-1.4	-7.7
Alanine	0.0	21.3	0.0	17.1	5.5	7.0	5.5	5.9	0.0950	0.0049	0.8083	0.0075	-1.0	2.0	0.0	1.8
Proline	0.0	-17.8	-15.8	-38.0	5.8	5.8	5.1	5.3	0.7105	0.0155	0.0084	0.0003	0.0	-1.6	-1.8	-3.2
Phosphocreatine	0.0	-18.9	-16.1	-42.3	4.3	4.7	8.2	8.5	0.8907	0.0040	0.0677	0.0027	0.0	-2.0	-1.1	-2.3
Phosphocholine	0.0	0.0	14.1	23.7	6.9	6.6	6.6	6.1	0.5552	0.9711	0.0746	0.0008	0.0	0.0	1.1	2.5
Glycine	0.0	42.8	16.6	48.8	2.2	2.5	3.1	3.3	1.0000	0.0000	0.0010	0.0000	0.0	12.5	2.6	11.6
Lactate	0.0	-17.5	0.0	-12.4	3.9	6.5	4.8	4.5	0.8733	0.0293	0.7729	0.0239	0.0	-1.4	0.0	-1.4
NAD+	0.0	11.9	0.0	16.7	6.9	5.8	6.3	6.5	0.3009	0.0738	0.1867	0.0196	-0.6	1.2	0.8	1.6
Formate	0.0	40.5	17.0	58.8	9.9	9.2	9.0	7.8	0.1970	0.0002	0.1129	0.0000	-0.7	3.2	0.9	6.3
Creatine	9.0	14.7	24.9	17.6	2.9	3.0	3.5	4.5	0.0143	0.0003	0.0003	0.0024	1.6	3.1	3.4	2.5
Cystathionine	10.9	0.0	12.1	-28.3	3.2	3.8	2.9	4.0	0.0096	0.3136	0.0031	0.0001	1.7	0.0	2.1	-3.4
2-Ketovaline	24.7	28.3	0.0	16.5	12.8	7.9	8.6	8.3	0.1401	0.0030	0.5099	0.0524	0.9	2.5	0.0	1.3
Valine	27.2	44.8	15.5	33.9	2.5	2.3	2.1	2.4	0.0000	0.0000	0.0000	0.0000	5.1	14.7	3.7	9.8
Histidine	29.6	37.5	11.8	22.5	3.1	4.7	5.2	3.5	0.0000	0.0000	0.0642	0.0000	4.4	5.7	1.1	4.1
Isoleucine	30.5	44.5	18.2	33.4	1.9	1.1	1.2	1.8	0.0000	0.0000	0.0000	0.0000	7.3	30.7	7.4	13.0
2-Oxoisoleucine	33.8	34.4	0.0	21.6	11.0	7.6	10.4	8.0	0.0266	0.0004	0.3293	0.0160	1.4	3.1	0.0	1.7
Leucine	39.9	60.3	33.4	54.2	4.0	2.5	3.3	3.7	0.0000	0.0000	0.0000	0.0000	4.4	20.3	4.7	11.7
Panthothenic	42.7	40.0	11.9	14.7	3.0	2.5	3.4	3.1	0.0000	0.0000	0.0086	0.0003	6.2	11.4	1.8	2.9
Phenylalanine	43.6	50.2	22.7	36.2	2.1	4.1	1.8	1.8	0.0000	0.0000	0.0000	0.0000	9.2	9.7	6.0	13.6
